# Ineffective Healthcare Technology Management in Benin’s Public Health Sector: The Perceptions of Key Actors and Their Ability to Address the Main Problems

**DOI:** 10.15171/ijhpm.2017.17

**Published:** 2017-02-20

**Authors:** P. Thierry Houngbo, Tjard De Cock Buning, Joske Bunders, Harry L. S. Coleman, Daton Medenou, Laurent Dakpanon, Marjolein Zweekhorst

**Affiliations:** ^1^ Ministry of Health, Cotonou, Republic of Benin.; ^2^ Athena Institute, Vrije Universiteit, Amsterdam, The Netherlands.; ^3^ Polytechnic School, University of Abomey-Calavi, Cotonou, Republic of Benin.

**Keywords:** Stakeholders, Healthcare Technology Management (HTM), Benin

## Abstract

**Background:** Low-income countries face many contextual challenges to manage healthcare technologies effectively, as the majority are imported and resources are constrained to a greater extent. Previous healthcare technology management (HTM) policies in Benin have failed to produce better quality of care for the population and costeffectiveness for the government. This study aims to identify and assess the main problems facing HTM in Benin’s public health sector, as well as the ability of key actors within the sector to address these problems.

**Methods:** We conducted 2 surveys in 117 selected health facilities. The first survey was based on 377 questionnaires and 259 interviews, and the second involved observation and group interviews at health facilities. The Temple-Bird Healthcare Technology Package System (TBHTPS), tailored to the context of Benin’s health system, was used as a conceptual framework.

**Results:** The findings of the first survey show that 85% of key actors in Benin’s HTM sector characterized the system as failing in components of the TBHTPS framework. Biomedical, clinical, healthcare technology engineers and technicians perceived problems most severely, followed by users of equipment, managers and hospital directors, international organization officers, local and foreign suppliers, and finally policy-makers, planners and administrators at the Ministry of Health (MoH). The 5 most important challenges to be addressed are policy, strategic management and planning, and technology needs assessment and selection – categorized as major enabling inputs (MEI) in HTM by the TBHTPS framework – and installation and commissioning, training and skill development and procurement, which are import and use activities (IUA). The ability of each key actor to address these problems (the degree of political or administrative power they possess) was inversely proportional to their perception of the severity of the problems. Observational data gathered during site visits described a different set of challenges including maintenance and repair, distribution, installation and commissioning, use and training and personnel skill development.

**Conclusion:** The lack of experiential and technical knowledge in policy development processes could underpin many of the continuing problems in Benin’s HTM system. Before solutions can be devised to these problems, it is necessary to investigate their root causes, and which problems are most amenable to policy development.

## Background

Healthcare technologies offer many benefits and have greatly enhanced the ability of health professionals to prevent, diagnose and treat diseases.^1^ They are a key component of a well-performing health system as they are the most abundant and widely used medical products in practice.^2^ The quality of healthcare delivery can be assessed along the STEEEP criteria (Safe, Timely, Effective, Efficient, Equitable, and Patient-centered),^3^ however, many developing countries in general, and Benin in particular, face problems satisfying these due to the unavailability, or limited availability of healthcare technologies. This study looks to elucidate the problems facing the management of healthcare technologies in Benin, by surveying the perceptions of actors within the sector.

Healthcare technology is defined as the application of organized knowledge and skills in the form of devices, medicine, vaccines, procedures and systems developed to solve health problems and improve the quality of lives.^1,4–9^ When used in this paper, the term “healthcare technology” means the different types of devices or equipment used in health facilities which encompasses medical equipment for clinical use, hospital furniture, vehicles, service supplies, plant, communication equipment, firefighting equipment, fixtures built into the building, office equipment, office furniture, training equipment, walking aids and workshop equipment.^4^

Healthcare technology management (HTM) is critical in developing countries due to many contextual challenges. Since about 95% of the healthcare technology used in these countries is imported,^10^ mismatches commonly occur because the technology development processes happening in developed countries has not accounted for the specific demands of the environment of placement. The process of transfer from high-income countries to non-producing countries leads to mismatches that consequently affect both the cost to the government and use of technology.^11-16^

Healthcare provision in Benin is organized through both the public and private health sectors. The public healthcare delivery system has been reorganized on the basis of a decentralization policy. It comprises of three hospital levels (the primary or zone hospital, the secondary or departmental hospital and the tertiary or university hospital) and many different health centers, with the zone hospital acting as the first referral hospital.^17^ Weaknesses in Benin’s public HTM sector that result in low efficiency, low cost-effectiveness and a low quality of overall community healthcare have been detailed in many technical reports by the Ministry of Health (MoH).^18-25^ Many facilities, especially primary and secondary hospitals, lack the basic technologies they require to provide quality care due to equipment being unavailable, inoperative, misused or inappropriate. The situation is most severe in the health facilities located in remote areas. This has far-reaching implications for the prevention and treatment of diseases and commonly leads to financial losses. Although huge financial resources are invested in the acquisition of devices, not enough attention is paid to their strategic management. While some healthcare equipment is donated, a significant proportion has been purchased with loans provided by bilateral and multilateral agencies and thus requires repayment.

Since 1995, many seminars, talks, workshops, discussions and surveys have been organized with diverse stakeholder groups participating, however, they have yet to be effectively translated into a successful policy.^18,22,25-29^ In fact, one study has shown that, in the wake of government’s attempts at reform, efficiency and productivity within zone hospitals actually decreased from 2003 to 2007.^30^ The first national maintenance policy for healthcare infrastructure, equipment and vehicles of 2002 was promising, but its implementation was halted due to a lack of political will and budgeted action plan. There have been some piecemeal attempts by the MoH, and some isolated, short-term actions of donors to support the development of maintenance and healthcare technology repair functions, but these have not proved to be comprehensive strategies to deal with this problem. Ineffective HTM has been reported in many sub-Saharan African countries, and other poor countries.^4,5,9,12,16,31-38^

The objective of this study is to identify and assess the main problems facing HTM in Benin’s public health sector and the ability of key actors within the sector, given the degree of political or administrative power they possess, to solve these problems. The main research question is: “What are the main problems facing HTM in Benin’s public health sector and how they could be they addressed according to the perceptions and power positions of each key actor?” The results and analysis of this study have been used to inform a recent policy developed for HTM in Benin, which was validated in April 2016.

The following sections describe the conceptual framework, methodological design, results, and then the findings of the study.

### Conceptual Framework

The Temple-Bird Healthcare Technology Package System (TBHTPS)^16^ has been used as a model framework for HTM in various developing countries. According to this framework, effective and efficient HTM could be seen as the product of a managerial process of 11 components, comprised of 5 major enabling inputs (MEI) and 6 import and use activities (IUA). These processes are steered by 4 key actors and influenced by a background context that takes into account 4 national parameters. When the framework was tailored to the specific context of Benin’s health system, the eleven components of the managerial process were broadened to 14 components. Furthermore, the number of key actors was defined as 6, the national context was extended to 5 parameters and an international context was added that comprises 2 parameters (Figure).

**Figure:**
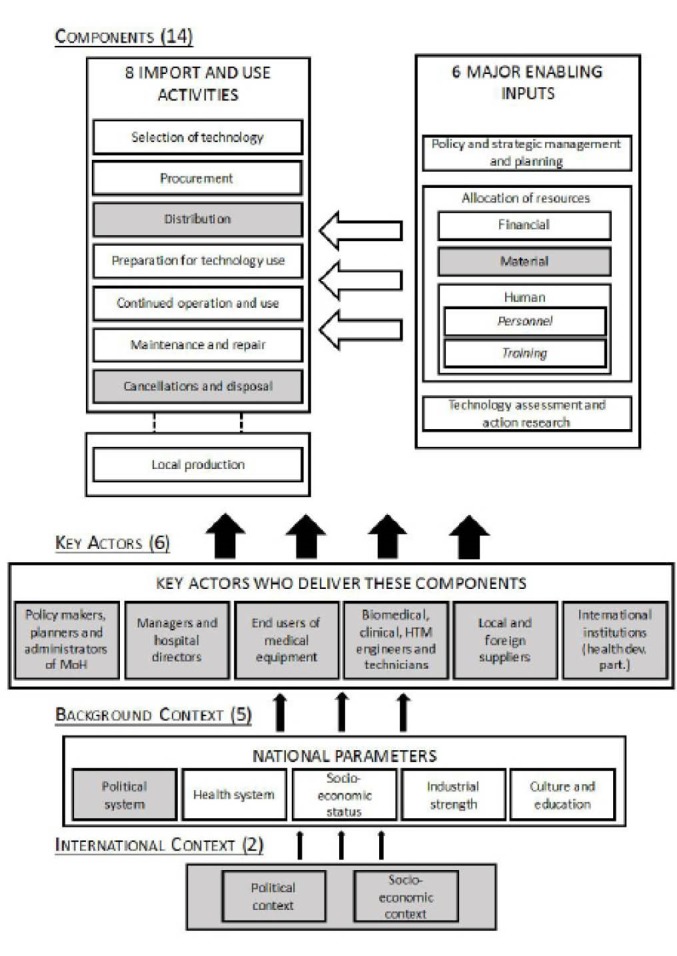
The Adapted Temple-Bird Healthcare Technology Package System (TBHTPS).^16^ Abbreviations: HTM, healthcare technology management; MoH, Ministry of Health.

The 14 components of the managerial process consist of 6 MEI (policy and strategic management and planning; allocation of financial resources; allocation of material resources; allocation of human resources, that comprises personnel and their training, and; technology assessment and action research) and 8 IUA (technology needs assessment and selection; procurement; distribution; preparation for technology use; continued operation and use; maintenance and repair; cancellation and disposal; and local production). The new element within the MEI is the *allocation of material resources* and the new elements within the IUA are *distribution* and *cancellation and disposal*. The allocation of material resources (other elements of infrastructure that are necessary for the supply, distribution and, maintenance and repair activities ie, vehicles) has been added to the TBHTPS framework as it represents a key process for successful HTM.^15^ Furthermore, the distribution of newly procured or donated health equipment in Benin is an activity that has received much criticism by the MoH.^22^ Cancellation and disposal of healthcare technologies remains a crucial issue in Benin as the absence of a policy defining these activities has resulted in a high proportion of obsolete equipment being left in many hospital wards.

The 6 types of actors who deliver these components are defined at a lower level of abstraction in this paper. Instead of using institutions as described in the TBHTPS model, here, they are represented by the professionals who are involved in HTM in Benin, including policy-makers, planners, and administrators at the MoH; hospital managers and directors; end users of medical equipment ie, medical doctors, nurses, midwives, lab technicians, X-ray and other imaging technicians; biomedical, clinical, or healthcare technology engineers and technicians; local and foreign suppliers; and international organization representatives ie, bilateral or multilateral health development partners.

The 5 national parameters comprising the background context are the political system, the health system, socio-economic status, industrial strength and the cultural and education context. Here, the political system has been added as a new parameter for the context of Benin since decision-making within its public administration is highly politicized (ie, the appointment of some civil servants to administrative positions is based on political considerations).

The background context itself is subjected to a complex international system that consists of at least 2 parameters: a political context and socio-economic context.^15^ As the majority of medical equipment procurement projects are financed by international health development partners (such as, bilateral or multilateral institutions), these international organizations also play key financial and technical roles in supporting the country’s national budget.

## Methods

A mixed-methods approach was used based on 2 surveys. The first survey addressed the main problems of ineffective HTM in Benin’s public health sector and the ways these could be tackled according to the perceptions of key actors. It was based on the use of written questionnaires and interviews, designed for data collection from a stratified selection of key HTM actors and public healthcare facilities, in 2010 and 2011. The second survey based on site visits was undertaken in 2012 to inventory the practical problems facing HTM in the same healthcare facilities.

Both the first and second survey made use of the adapted TBHTPS framework. The first survey used the MEI-IUA of the framework to formulate questions relating to problems in each component. Additionally, the responses of the first survey, both the questionnaires and interviews, were coded and categorized based on the MEI-IUA. The second survey used the MEI-IUA to categorize problems identified in healthcare facilities during site visits. The results of both surveys attempt to provide an exhaustive list of problems in HTM in Benin. Analyzing how problems were identified and prioritized by different actor groups was also of interest.

### Selection of Participants and Healthcare Facilities

The relevant stakeholders involved in the first and second survey consisted of key HTM actors (N = 5302) and public healthcare facilities (N = 787). Both actors and healthcare facilities were surveyed during the first survey, whilst only healthcare facilities were surveyed during the second. Key HTM actors include policy-makers, planners, and administrators at the MoH; hospital managers and directors; end users of medical equipment; biomedical, clinical, or healthcare technology engineers and technicians; local and foreign suppliers; and international organization representatives ie, bilateral or multilateral health development partners. The surveyed healthcare facilities include health centers and hospitals. Using the statistical determination of sample size based on precision rate (±10) and confidence level (95%) for a finite population,^39^ the theoretical sample sizes for the survey were 244 key HTM actors, for whom questionnaires and interviews were designed, and 117 public healthcare facilities. Because the sampling frames from which the samples were drawn did not constitute a homogenous group, stratified sampling has been used in order to achieve fair representation. Furthermore, in order to obtain sufficient responses, 500 questionnaires were distributed and more than 259 interviews were conducted. Table 1 presents the sample of the questionnaire and interview survey, whereas Table 2 shows the sample of the site visits.

**Table 1:** Origins of the Data, Including the Selected Healthcare Facilities and the Actors Who Participated in Interviews or Questionnaires

**Healthcare Facility**	**Policy-Makers, Planners, Administrators at MoH**				**Hospital Directors and Managers**				**Users of Equipment**				**Biomedical, Clinical, Health Technology Engineers and Technicians**				**Local and Foreign Suppliers**				**International Organization Representatives**			
		**T**	**TSS**	**Q**	**I**	**T**	**TSS**	**Q**	**I**	**T**	**TSS**	**Q**	**I**	**T**	**TSS**	**Q**	**I**	**T**	**TSS**	**Q**	**I**	**T**	**TSS**	**Q**	**I**
Central	MS	36	1	18 (19)	12	10				202	9	4 (4)	1	12	1	7 (10)	8	35	2	15 (20)	11	22	1	6 (10)	5
	CNHU					4		2 (2)	1	528	24	19 (22)	10	4		1 (2)	1								
	CNPP					2		1 (1)	1	16	1	4 (6)	3												
	CNP					2		1 (1)	1	7	1	2 (4)	1												
	CNG					2				12	1	0 (3)	1												
	HOMEL					3		1 (1)	1	92	4	14 (18)	7	1		1 (1)	1								
	LN									17	1	6 (8)	6												
Intermediate	DDS	6	1	5 (6)	2	6		3 (6)	1	36	2			7	1	2 (4)	4								
	CHD					10	2	7 (10)	6	412	19	68 (75)	24	5		2 (4)	3								
	CIPEC					3				36	2	2 (6)	2												
	CETAL					4				48	2	3 (6)	2												
	CUB-AP					2				22	1	2 (4)	1												
	CPP-A					2				12	1	1 (2)	1												
Peripheral	C-ZS					8		7 (12)	9	34	2	13 (18)	9												
	HZ					38	2	10 (12)	10	652	30	94 (115)	65	5		1 (4)	4								
	CSC									839	39	20 (28)	17												
	CSA									1869	82	31 (42)	22												
	CASES									48	2	1 (2)	1												
	CDT									98	5	0 (4)	1												
	UVS									6	1	0 (2)	1												
	DI/MI									87	4	3 (6)	3												
Total	42	2	23 (25)	14	96	4	32 (45)	30	5073	233	287 (375)	178	34	2	14 (25)	21	35	2	15 (20)	11	22	1	6 (10)	5

Abbreviations: T, total number in Benin; TSS, theoretical sample size; Q, questionnaires; I, interviews; MS, Ministry of Health; CNHU, National Teaching Hospital; CNPP, National Tuberculosis Treatment Center; CNP, National Hospital of Psychiatry; CNG, National Hospital of Gerontology; HOMEL, National Maternal and Childrens’ Hospital; LN, National Research Hospital; DDS, Health Department Directorate; CHD, Departmental Hospital; CIPEC, Health Information and Advisory Center; CETAL, Leprosy Treatment Center; CUB-AP, Buruli Ulcer Treatment Centers; CPP-A, Tuberculosis Treatment Center of Akron; C-ZS, Health Zone Committee; HZ, Zone Hospital; CSC, Commune (District) Health Center; CSA, Arrondissement (Municipality) Health Center; CASES, Solidarity and Health Center; CDT, Tuberculosis Detection Center; UVS, Village Health Unit; DI/MI, Remote Dispensary and Maternity Units. Note: Brackets indicate total returned questionnaires, numbers outside brackets indicates how many were used in the analysis.

**Table 2:** Overview of the Healthcare Facilities Visited During Site Visits

**Healthcare Facility**	**Total**	**TSS**
Central	MS		
	CNHU	2	1
	CNPP	1	1
	CNP	1	1
	CNG	1	1
	HOMEL	1	1
	LN	1	1
Intermediate	DDS		
	CHD	5	1
	CIPEC	6	1
	CETAL	8	1
	CUB-AP	2	1
	CPP-A	1	1
Peripheral	C-ZS		
	HZ	27	4
	CSC	77	11
	CSA	487	66
	CASES	24	4
	CDT	50	7
	UVS	6	1
	DI/MI	87	13
Total	787	117

Abbreviations: T, total number in Benin; TSS, theoretical sample size; Q, questionnaires; I, interviews; MS, Ministry of Health; CNHU, National Teaching Hospital; CNPP, National Tuberculosis Treatment Center; CNP, National Hospital of Psychiatry; CNG, National Hospital of Gerontology; HOMEL, National Maternal and Childrens’ Hospital; LN, National Research Hospital; DDS, Health Department Directorate; CHD, Departmental Hospital; CIPEC, Health Information and Advisory Center; CETAL, Leprosy Treatment Center; CUB-AP, Buruli Ulcer Treatment Centers; CPP-A, Tuberculosis Treatment Center of Akron; C-ZS, Health Zone Committee; HZ, Zone Hospital; CSC, Commune (District) Health Center; CSA, Arrondissement (Municipality) Health Center; CASES, Solidarity and Health Center; CDT, Tuberculosis Detection Center; UVS, Village Health Unit; DI/MI, Remote Dispensary and Maternity Units.

### First Survey

#### Questionnaires and Interviews

Six types of questionnaires were designed for the 6 types of key actors in HTM in Benin. The questions concerned 10 MEI-IUA of the adapted TBHTPS framework model instead of 14. Questions concerning “technology assessment and action research” and “local production” were not included as these 2 activities are not yet adequately established in Benin’s HTM sector. Furthermore, 4 MEI (allocation of financial, material, personnel and training resources) were reorganized into 2 thematic groups: “budgeting and financing” and “training and personnel skill development.” The resulting 10 MEI-IUA, therefore, were: (1) policy and strategic management and planning; (2) budgeting and financing; (3) technology needs assessment and selection; (4) procurement; (5) distribution; (6) preparation for technology use; (7) installation and commissioning; (8) training and personnel skill development, continued operation and use; (9) maintenance and repair; and (10) cancellation and disposal. At least 3 questions were asked on each of these 10 MEI-IUA. The questions related to understanding each actor’s perceptions on the main problems facing HTM in Benin and the relative degree of political or administrative power they possessed pertaining to solving these problems. Local and foreign suppliers of healthcare technologies, however, were not asked about MEI-IUA 1 (policy and strategic management and planning) or MEI-IUA 3 (technology needs assessment and selection) as it was not relevant to their professional remit. Questions asked to international organization representatives focused on MEI-IUA 4 and 9. In an attempt to make explicit the entirety of problems in HTM, the majority of questions required open responses except for qualifying or demographic questions that were structured. The questionnaires were distributed from December 2010 to February 2011 and 2 weeks were allotted to allow the return of questionnaires or collection by the research team. Contact information was collected in the questionnaire for prospective clarification in the case of errors and omissions in respondent’s answers.

Face-to-face semi-structured interviews (n = 259) took place with a stratified sample of key actors at the MoH, its decentralized departments and in 117 selected healthcare facilities from December 2010 to May 2011. The majority of the interviewees were also respondents of the questionnaire. Two interview teams were set up in order to conduct the interviews. Each team consisted of a main interviewer and 2 technicians who recorded or took written notes. The interview teams visited respondents at their place of work and asked them to provide their perceptions on the 10 MEI-IUA of the adapted TBHTPS framework. Specifically, questions were asked regarding the main problems encountered during their work in Benin’s HTM system and their perception of the power possessed by other actors to solve these problems. Finally, every respondent was invited to name additional weaknesses or problems in the current HTM system. Each interview lasted 30-60 minutes. With approval of some respondents (the users of equipment), 47 interviews were recorded and transcribed. A report of these interviews was returned to the respondents for feedback and validation. Written notes were taken during the 212 interviews that were not recorded, and these were also written into reports and validated by respondents.

#### Data Analysis

Out of the 500 questionnaires distributed to the sample of key HTM actors, 377 (75.4%) were returned (see Table 1). Errors made by respondents when completing the questionnaire related to the type and size of healthcare facility, institution, or company at which they worked, and some demographic information was completed or corrected by the research team after contact with the informant. Of the returned questionnaires, not all actors provided answers to all elements of the MEI-IUA surveyed, which resulted in 372 questionnaires being used in the analysis. Open coding was done manually and cross checked by members of the research team. Twelve sub-topics emerged that were categorized within the ten MEI-IUA of the adapted TBHTPS framework (Table 3). For example, code 1.1 (“ineffective and inefficient HTM system”) represents a sub-topic that can be categorized within MEI-IUA 1; code 2.1 (“loss of scarce resources in the importation of inappropriate medical devices”) and code 2.2 (“inadequate financial resource allocation for the life cycle cost [LCC] of the equipment”) can be categorized within MEI-IUA 2. Each sub-topic represents the main problem(s) related to or, as a result of, the MEI-IUA of HTM in Benin.

**Table 3:** List of the 12 Sub-topics That Emerged as a Result of Interview and Questionnaire Coding

**No**	**Code**	**Problems**
1		Policy and strategic management and planning
	1.1	Ineffective and inefficient HTM system
2		Budgeting and financing
	2.1	Loss of scarce resources in the importation of inappropriate medical devices
	2.2	Inadequate financial resource allocation for the LCC of the equipment
3		Technology needs assessment and selection
	3.1	Inappropriately selected equipment
4		Procurement
	4.1	High acquisition prices and origin of the equipment
	4.2	Inappropriate and unsuitable equipment procured
5		Distribution
	5.1	Unequal and inappropriate distribution (site, size, capacity of the health facility) of procured or donated equipment
6		Installation and commissioning
	6.1	Uninstalled and uncommissioned equipment due to inadequate architectural design and technical (power, water and gas supplies) capacities
7		Training and skill development
	7.1	Low rate of equipment availability
8		Operation and safety
	8.1	High proportion of hazardous and unsafe equipment used (ie, equipment lacking regular safety and performance checks with the ability to cause harm to the patient or user)
9		Maintenance and repair
	9.1	High proportion of unavailable equipment
10		Decommissioning and disposal
	10.1	High proportion of obsolete equipment in many healthcare facility wards

Abbreviations: LCC, life cycle costs; HTM, healthcare technology management. Note: Each sub-topic relates to a problem in one of the import and use activities, or major enabling inputs from the adapted Temple-Bird framework.

The 47 semi-structured interviews that were recorded and the 212 interviews, during which notes were taken, were transcribed and coded in the same way. After transcription, only 255 of the 259 interviews could be analyzed due to missing information concerning the MEI-IUA. Contrary to the questionnaires, only 10 sub-topics emerged from the 10 MEI-IUA based on the responses given in interviews. Each of the sub-topics that emerged represents a main problem related to a MEI-IUA of HTM in Benin. The interviews did not reproduce codes 2.1 (“loss of scarce resources in the importation of inappropriate medical devices”) and 4.2 (“inappropriate and unsuitable procured equipment”) that had been identified during the questionnaires as main problems in Benin’s HTM sector. Answers or information related to these codes were adequately represented by codes 4.1 (“high acquisition cost and origin of the equipment”) and 3.1 (“inappropriate selected equipment”) in the responses of interviewees.

### Second Survey

#### Site Visits

Site visits were undertaken in the stratified sample of 117 healthcare facilities from February to March 2012 to inspect the placing of the equipment in these facilities and to describe their inherent problems, such as the causes of ineffective equipment use and maintenance. During the visits, observation notes were taken based on the adapted TBHTPS framework. At the end of each visit, a group interview (thirty minutes to one hour) was organized with staff of the healthcare facility to prioritize HTM problems for future policy actions, according to their severity. An overview of the healthcare facilities visited is presented in Table 2.

#### Data Analysis

For the analysis of the data of the site visits, the main issues affecting HTM in Benin were grouped into 24 factors according the adapted TBHTPS framework. A characterization of each of the factors was expressed in a qualitative 8-point scale (comprised of 4 positive and 4 negative points). A positive rating could be **(++++)** excellent, **(+++)** very good, **(++)** good, or **(+)** sufficient. Conversely, a negative rating could be **(-)** mediocre, **(--)** bad, **(---)** very bad, or **(----)** extremely bad. Based on the notes taken, inspection data and the local discussions organized, the main problems in HTM in Benin were ranked.

## Results

### First Survey

#### Perceptions of Key Actors Towards the Main Problems

Tables 4 and 5 show that the 12 main problems facing HTM in Benin’s public health sector were identified by 85% (3791/4464) of questionnaire respondents, 86% (2197/2548) of interviewees, and thus 85% (5988/7012) of all respondents. There is no significant difference between the questionnaire and interview findings (χ^2^= 0.21, *P* > .05). The data collection technique (using interviews or questionnaires) had no effect on the severity with which problems were perceived by different actor groups. Table 6 shows the order in which actor groups perceived problems most severely, namely: biomedical, clinical, healthcare technology engineers or technicians= 100% (14/14) for questionnaire respondents, 95% (20/21) for interviewees, 97% for both (34/35); users of equipment in healthcare facilities= 91% (261/287), 93% (165/178) and 92% (426/465), respectively; hospital managers and directors= 75% (24/32), 77% (23/30) and 77% (48/62), respectively: international organization representatives= 67% (4/6), 60% (3/5) and 64% (7/11), respectively; local and foreign suppliers= 47% (7/15), 55% (6/11) and 50% (13/26), respectively, and; policy-makers, planners, administrators at the MoH= 35% (8/23), 36% (5/14) and 35% (13/37), respectively.

**Table 4:** Findings of the Questionnaire and Interview Surveys Showing How HTM Problem Sub-topics 1.1-5.1 Were Prioritized by Each Actor Group

**Key Actor Group**	**Code**																								
	**1.1**			**2.1**			**2.2**				**3.1**				**4.1**			**4.2**				**5.1**			
	**Q**	%	**I**	%	**Q**	%	**Q**	%	**I**	%	**Q**	%	**I**	%	**Q**	%	**I**	%	**Q**	%	**Q**	%	**I**	%
(iv)	14 (14)	100	20 (21)	95	14 (14)	100	14 (14)	100	21 (21)	100	13 (14)	93	19 (21)	90	12 (14)	86	18 (21)	86	13 (14)	93	14 (14)	100	20 (21)	95
(iii)	284 (287)	99	172 (178)	97	267 (287)	93	235 (287)	82	157 (178)	88	284 (287)	99	172 (178)	97	264 (287)	92	172 (178)	97	241 (287)	84	187 (287)	65	120 (178)	67
(ii)	31 (32)	97	28 (30)	93	31 (32)	97	31 (32)	97	29 (30)	97	27 (32)	84	27 (30)	90	21 (32)	66	25 (30)	83	23 (32)	72	14 (32)	44	14 (30)	47
(vi)															5 (6)	83	4 (5)	80	3 (6)	50	1 (6)	17	1 (5)	20
(v)					4 (15)	27	14 (15)	93	10 (11)	91					9 (15)	60	7 (11)	64	6 (15)	40	11 (15)	73	8 (11)	73
(i)	14 (23)	61	9 (14)	64	12 (23)	52	10 (23)	43	7 (14)	50	9 (23)	39	6 (14)	43	14 (23)	61	9 (14)	64	10 (23)	43	12 (23)	52	8 (14)	57
Total	343 (356)	96	229 (243)	94			304 (371)	82	224 (254)	88	333 (356)	94	224 (243)	92	325 (377)	86	235 (259)	91			239 (377)	63	171 (259)	66
		572 (599)			328 (371)	88		528 (625)			557 (599)				560 (636)			296 (377)	79		410 (636)			
%		95.49						84.48				92.99				88.05						64.47		
Rank		1						8				2				5						10		

Abbreviation: HTM, healthcare technology management.

**Table 5:** Findings of the Questionnaire and Interview Surveys Showing How HTM Problem Sub-topics 6.1-10.1 Were Prioritized by Each Actor Group

**Key Actor Group**	**Code**																				**Total/Mean**			
	**6.1**				**7.1**				**8.1**				**9.1**				**10.1**							
	**Q**	%	**I**	%	**Q**	%	**I**	%	**Q**	%	**I**	%	**Q**	%	**I**	%	**Q**	%	**I**	%	**Q**	%	**I**	%
(iv)	14 (14)	100	21 (21)	100	14 (14)	100	21 (21)	100	14 (14)	100	21 (21)	100	13 (14)	93	21 (21)	100	14 (14)	100	21 (21)	100	163 (168)	97	203 (210)	97
(iii)	270 (287)	94	171 (178)	96	284 (287)	99	172 (178)	97	273 (287)	95	167 (178)	94	281 (287)	98	172 (178)	97	267 (287)	96	171 (178)	96	3137 (3444)	91	1646 (1780)	92
(ii)	26 (32)	81	25 (30)	83	25 (32)	78	23 (30)	77	15 (32)	47	15 (30)	50	24 (32)	75	22 (30)	73	25 (32)	78	24 (30)	80	293 (384)	76	232 (300)	77
(vi)	2 (6)	33	2 (5)	40	6 (6)	100	4 (5)	80	2 (6)	33	2 (5)	40	6 (6)	100	4 (5)	80					25 (42)	60	17 (30)	57
(v)	15 (15)	100	10 (11)	91	5 (15)	33	4 (11)	36	2 (15)	13	2 (11)	18	3 (15)	20	2 (11)	18	4 (15)	27	3 (11)	27	73 (150)	49	46 (88)	52
(i)	7 (23)	30	5 (14)	36	4 (23)	17	3 (14)	21	3 (23)	13	2 (14)	14	3 (23)	13	2 (14)	14	2 (23)	9	2 (14)	14	100 (276)	36	53 (140)	38
Total	334 (377)	89	234 (259)	90	338 (377)	90	227 (259)	88	309 (377)	82	209 (259)	81	330 (377)	88	223 (259)	86	312 (371)	84	221 (254)	87	3791 (4464)	85	2197 (2548)	86
	568 (636)				565 (636)				518 (636)				553 (636)				533 (625)				5988 (7012)			
%	89.31				88.84				81.45				86.95				85.28				85.40			
Rank	3				4				9				6				7							

Abbreviation: HTM, healthcare technology management. Legend: (*i*) Policy-makers, planners, and administrators at the MoH; (*ii*) hospital managers and directors; (*iii*) end users of medical equipment; (*iv*) biomedical, clinical and healthcare technology engineers and technicians; (v) local and foreign suppliers; (*vi*) international organization representatives. 1.1 Ineffective and inefficient HTM system; 2.1 Loss of scarce resources in the importation of inappropriate medical devices; 2.2 Inadequate financial resource allocations for the life-cycle costs (LCC) of equipment; 3.1 Inappropriately selected equipment; 4.1 High acquisition prices and origin of equipment; 4.2 Inappropriate and unsuitable equipment procured; 5.1 Unequal and inappropriate distribution (site, size, capacity of the health facility) of procured or donated equipment; Uninstalled and un-commissioned equipment due to inadequate architectural design and technical (power, water and gas supplies) capacities; 7.1 Low rate of equipment availability; 8.1 High proportion of hazardous and unsafe equipment used; 9.1 High proportion of unavailable equipment; 10.1 High proportion of obsolete equipment in many healthcare facility wards. Note: Brackets indicate total returned questionnaires, numbers outside brackets indicates how many were used in the analysis.

**Table 6:** Perceptions of the Severity of Problems of Each Actor Group, as a Result of Summing Individually Rated Problems

**Key Actor Group**	**Questionnaires**		**Interviews**		**Questionnaires + Interviews**		**Rank**
	**No.**	**%**	**No.**	**%**	**No.**	**%**	
Biomedical, clinical, healthcare technology engineers and technicians	163 (168)	97.02	203 (210)	96.67	366 (378)	96.83	1
Users of equipment in healthcare facilities	3137 (3444)	91.09	1646 (1780)	92.47	4783 (5224)	91.56	2
Managers and hospital directors	293 (384)	76.30	232 (300)	77.33	525 (684)	76.75	3
International organization representatives	25 (42)	59.52	17 (30)	56.67	42 (72)	58.33	4
Local and foreign suppliers	73 (150)	48.67	46 (88)	52.27	119 (238)	50.00	5
Policy makers, planners, administrators at the MoH	100 (276)	36.23	53 (140)	37.86	153 (416)	36.78	6
Total/mean	3791 (4464)	84.92	2197 (2548)	86.22	5988 (7012)	85.40	

Abbreviations: LCC, life cycle costs; MoH, Ministry of Health.

### Prioritized Problems of the 6 Key Actors

Tables 7, 8 and 9 present how the problems identified in HTM were prioritized (based on their severity/priority for action) by all actors when using questionnaires, interviews or both, respectively. Among the 12 sub-topics identified from questionnaire responses, the 5 most crucial problems that require action, based on the severity of actors’ perceptions were: (*i*) “ineffective and inefficient HTM system” (code 1.1); (*ii*) “inappropriately selected equipment” (code 3.1); (*iii*) “low rate of equipment availability” (code 7.1); (*iv*) “uninstalled and un-commissioned equipment due to inadequate architectural (space and design) and technical (electrical power, water and gas supplies) requirements” (code 6.1); (*v*) “loss of scarce resources in the importation of inappropriate medical devices (code 2.1); and (*vi*) “high proportion of unavailable equipment” (code 9.1) (Table 7). The 5 main problems prioritized by interview respondents were: (*i*) “ineffective and inefficient HTM system” (code 1.1); (*ii*) “inappropriately selected equipment” (code 3.1); (*iii*) “high acquisition costs and origin of the equipment” (code 4.1); (*iv*) “uninstalled and un-commissioned equipment due to inadequate architectural (space and design) and technical (electrical power, water and gas supplies) requirements” (code 6.1); (*v*) “low rate of equipment availability” (code 7.1); and (*vi*) “inadequate financial resource allocation for the LCCs of equipment” (code 2.2.) (Table 8). When combining the results of questionnaires and interviews, it was found that among the 12 sub-topics identified (except sub-topics 2.1 and 4.2, which were not investigated during interviews), the 5 main HTM problems were: (*i*) “ineffective and inefficient HTM system” (code 1.1); (*ii*) “inappropriately selected equipment” (code 3.1); (*iii*) “uninstalled and commissioned equipment due to inadequate architectural (space and design) and technical (power, water and gas supplies) requirements” (code 6.1); (*iv*) “low rate of equipment availability” (code 7.1); and (*v*) “high acquisition costs and origin of the equipment” (code 4.1) (Table 9). These 5 challenges belonged to the policy, strategic management and planning; technology needs assessment and selection; installation and commissioning; training and skill development; and procurement MEI-IUA of the adapted TBHTPS framework.

**Table 7:** Prioritization of Problems Based on Questionnaire Responses, as a Result of Pooling the Perceptions of Every Actor Group for Each Problem

**Code**	**No.**	**%**	**Rank**
1.1	Ineffective and inefficient HTM system	343 (356)	96.35	1
3.1	Inappropriately selected equipment	333 (356)	93.54	2
7.1	Low rate of equipment availability	338 (377)	89.66	3
9.1	High proportion of unavailable equipment	330 (370)	89.19	4
6.1	Uninstalled and un-commissioned equipment due to inadequate architectural design and technical (power, water and gas supplies) capacities	334 (377)	88.59	5
2.1	Loss of scarce resources in the importation of inappropriate medical devices	328 (371)	88.41	6
4.1	High acquisition prices and origin of the equipment	325 (377)	86.21	7
10.1	High proportion of obsolete equipment in many healthcare facility wards	312 (371)	84.10	8
8.1	High proportion of hazardous and unsafe equipment used	309 (377)	81.96	9
2.2	Inadequate financial resource allocation for the life cycle costs (LCC) of equipment	304 (371)	81.94	10
4.2	Inappropriate and unsuitable equipment procured	296 (377)	78.51	11
5.1	Unequal and inappropriate distribution (site, size, capacity of the health facility) of procured or donated equipment	239 (377)	63.40	12

Abbreviations: LCC, life cycle costs; HTM, healthcare technology management.

**Table 8:** Prioritization of Problems Based on Interview Responses, as a Result of Pooling the Perceptions of Every Actor Group for Each Problem

**Code**	**No.**	**%**	**Rank**
1.1	Ineffective and inefficient HTM system	229 (243)	94.24	1
3.1	Inappropriately selected equipment	224 (243)	92.18	2
4.1	High acquisition prices and origin of the equipment	235 (259)	90.73	3
6.1	Uninstalled and uncommissioned equipment due to inadequate architectural design and technical (power, water and gas supplies) capacities	234 (259)	90.35	4
2.2	Inadequate financial resource allocation for the LCC of equipment	224 (254)	88.19	5
7.1	Low rate of equipment availability	227 (259)	87.64	6
10.1	High proportion of obsolete equipment in many healthcare facility wards	221 (254)	87.01	7
9.1	High proportion of unavailable equipment	223 (259)	86.10	8
8.1	High proportion of hazardous and unsafe equipment used	209 (259)	80.69	9
5.1	Unequal and inappropriate distribution (site, size, capacity of the health facility) of procured or donated equipment	171 (259)	66.02	10
2.1	Loss of scarce resources in the importation of inappropriate medical devices			
4.2	Inappropriate and unsuitable equipment procured			

Abbreviations: LCC, life cycle costs; HTM, healthcare technology management.

**Table 9:** Prioritization of Problems Based on Interview and Questionnaire Responses Combined

**Code**	**No.**	**%**	**Rank**
3.1	Inappropriately selected equipment	557 (599)	92.99	1
1.1	Ineffective and inefficient HTM system	542 (599)	90.48	2
6.1	Uninstalled and uncommissioned equipment due to inadequate architectural design and technical (power, water and gas supplies) capacities	568 (636)	89.31	3
7.1	Low rate of equipment availability	565 (636)	88.84	4
4.1	High acquisition prices and origin of the equipment	560 (636)	88.05	5
9.1	High proportion of unavailable equipment	553 (629)	85.28	6
10.1	High proportion of obsolete equipment in many healthcare facility wards	533 (625)	85.28	7
2.2	Inadequate financial resource allocation for the LCC of equipment	528 (625)	84.48	8
8.1	High proportion of hazardous and unsafe equipment used	518 (636)	81.45	9
5.1	Unequal and inappropriate distribution (site, size, capacity of the health facility) of procured or donated equipment	410 (636)	64.47	10
2.1	Loss of scarce resources in the importation of inappropriate medical devices			
4.2	Inappropriate and unsuitable equipment procured			

Abbreviations: LCC, life cycle costs; HTM, healthcare technology management.

### Power Positions of Key Actors to Solve Healthcare Technology Management Problems

Table 10 presents the degree of power (political or administrative) attributed by actors to each other, relative to solving problems in HTM. Converse to the perceptions of problems held by key actors, the group of actors ranked highest, regarding their ability to solve problems, was the policy-makers, planners and administrators at the MoH (acknowledged by 39% [245/636] of questionnaire respondents and interviewees), followed by the local and foreign suppliers (21% [57/636]), international organization representatives (16% [101/636]), hospital directors and managers (10% [64/636]), users of equipment in healthcare facilities (9% [26/636]), and finally the biomedical, clinical and healthcare technology engineers and technicians (5% [10/636]). Table 11 shows the professional backgrounds of past and present Directors of the Department of Infrastructure, Equipment and Maintenance (DIEM), which is responsible for HTM in Benin. This position has been predominated by civil engineering specialists.

**Table 10:** Degree of Administrative or Political Power Possessed by Each Actor That Is Relative to Solving HTM Problems, as Identified by Other Actor Groups

**Key Actor Group**	**Questionnaires**		**Interviews**		**Questionnaires + Interviews**		**Rank**
	**No.**	**%**	**No.**	**%**	**No.**	**%**	
Policy-makers, planners, administrators at the MoH	147 (377)	38.99	98 (259)	37.84	245 (636**)**	38.52	**1**
Local and foreign suppliers	79 (377)	20.95	57 (259)	22.01	136 (636**)**	21.38	**2**
International organization representatives	57 (377)	15.12	44 (259)	16.99	101 (636**)**	15.88	**3**
Managers and hospital directors	41 (377)	10.88	23 (259**)**	8.88	64 (636**)**	10.06	**4**
Users of equipment in healthcare facilities	30 (377)	7.96	26 (259**)**	10.04	56 (636**)**	8.81	**5**
Biomedical, clinical, healthcare technology engineers and technicians	23 (377)	6.10	10 (259**)**	3.86	33 (636**)**	5.19	**6**

Abbreviations: HTM, healthcare technology management; MoH, Ministry of Health.

**Table 11:** Professional Background of the Director of the DIEM in Benin’s MoH from 1990 to 2011

**Year**	**1990-1993**	**1993-1996**	**1996-2000**	**2000-2002**	**2002-2004**	**2004-2006**	**2006-2011**	**2011-2016**	**2016-**
Professional background	Civil engineer	Civil engineer	Civil engineer	Civil engineer	Senior technician	Civil engineer	Doctor	Dentist	Biomedical engineer

Abbreviations: DIEM, the Department of infrastructure, equipment and maintenance; MoH, Ministry of Health.

### Problems Identified During Site Visits

The most important findings of the site visits are summarized in Table 12. The site visits revealed weaknesses in each of the 10 HTM components of the adapted TBHTPS framework, which have contributed to poor healthcare delivery. In order of decreasing importance, these were: (1) maintenance and repair, (2) distribution, (3) installation and commissioning, (4) use, (5) training and personnel skill development, (6) cancellation and disposal, (7) technology needs assessment and selection, (8) budgeting and financing, (9) policy, strategic management and planning, and (10) procurement. It was found that the main problem of Benin’s HTM is related to the maintenance and repair of the equipment. The weakest component of HTM identified during site visits was “Equipment’s operational performance,” that encompasses the low rate of equipment availability and poor technical performance of equipment. Although this is a direct product of ineffective maintenance and repair capacities, the availability of equipment, and it’s functioning, is also impacted by weaknesses in the other nine import and use capacities, that could underpin its position as the weakest identified HTM component. HTM issues were most evident at the intermediate and peripheral health facility levels, particularly in departmental and zone hospitals where maintenance structures and practices were lacking compared to the central level hospital. Indeed, the availability and the technical performance of healthcare equipment decreased with the size of the healthcare facility. In all visited health facilities, the proportion of medical devices out of operation was estimated to be 48% (1360/2822); 65% (859/1326) in the central hospital, 40% (264/658) in the departmental hospitals, 30% (144/483) in the zone hospitals and 26% (93/355) in health centers (see Table 13). Policy, strategic management and planning, and procurement were identified as less of a problem during site visits as the main actors involved in HTM capacities in health facilities – end users of the equipment, maintenance technicians, and hospital managers and directors – have little influence or insight into the planning and acquisition process that occurs at the MoH level.

**Table 12:** Factors Affecting Components of HTM in the 117 Healthcare Facilities Visited

**Rank**	**Factors Affecting HTM in Benin**	**Healthcare Facilities**		
		**Central**	**Intermediate**	**Peripheral**
1st	Maintenance and repair			
	Equipment’s operational performance	++	-	--
	Use of unhazardous and safe equipment	+	-	-
	Technical assessment of equipment	-	--	--
	Availability of service and user manuals	-	--	--
	Availability of after-sale service	-	--	--
	Availability of equipment spare parts	-	--	--
	Availability of maintenance technicians	-	--	---
	Availability of maintenance tools	-	--	--
	Equipment manufactured in same country	---	---	---
2nd	Distribution			
	Equal and appropriate distribution (site, size, capacity of the health facility) of procured or donated equipment	+	-	--
	Appropriate technology for the site, size, capacity of health facility	+	-	--
3rd	Installation and commissioning			
	Installed equipment with adequate architectural design and technical (power, water and gas supplies) capacities	+	-	--
	Availability and quality of electric power	-	-	-
4th	Use			
	Effective use of equipment	+	+	-
	Availability of equipment user manuals	-	-	--
5th	Training and personnel skill development			
	Regular training of equipment users and maintenance technicians	-	-	--
6th	Cancellation and disposal			
	Absence of obsolete equipment in healthcare facility wards	+	-	-
7th	Technology needs assessment and selection			
	Implementation of computerized asset management for needs assessments	+	-	-
8th	Budgeting and financing			
	Availability of resources (financial, material and human) for maintenance	+	-	--
	Availability of annual maintenance budget	-	-	-
9th	Policy and strategic management and planning			
	Homogeneity of equipment makes and models	--	---	---
	Maintenance planning	+	-	-
10th	Procurement			
	Involvement of equipment users in acquisition processes	+	-	-
	Availability of information on equipment acquisition prices	-	--	--

Abbreviation: HTM, healthcare technology management.

**Table 13:** Proportion of Equipment out of Operation in Selected Health Facilities

**Health Facility**	**Equipment Availability**		
	**Total Number of Selected Equipment**	**Number of Equipment out of Operation**	**Proportion of Equipment out of Operation**
Central hospital (2)	1326	859	859/1326 (64.78%)
Departmental hospitals (2)	658	264	264/658 (40.12%)
Zone hospitals (4)	483	144	144/483 (29.81%)
Health centers (10)	355	93	93/355 (26.19%)
All selected health facilities	2822	1360	1360/2822 (48.19%)

## Discussion

This section discusses the findings, presents the limitations of the research, and indicates further research opportunities to support or answer the questions raised by this study.

When exploring the perceptions of key actors regarding the HTM issues identified, there was no significant difference between the results of questionnaires and interviews (triangulation). The results show that biomedical, clinical and healthcare technology engineers and technicians, the users of equipment in healthcare facilities and the hospital managers and directors possess the greatest concern for HTM, and thus face more problems with medical device management and maintenance than other actor groups. These actors most frequently come into contact with equipment, and any failure that results from their ineffective use or maintenance has negative implications for the quality of the healthcare services provided. They are also more socially and legally accountable (at different degrees) than other actors for patient injuries or accidents that happen in healthcare facilities. Biomedical, clinical and healthcare technology engineers and technicians are also responsible for the maintenance of equipment, and are appraised in line with maintenance unit performance.

The low priority given by respondents to healthcare technology distribution problems conflicts with the findings of the site visits where it was the second-highest ranking problem. The unequal and inappropriate distribution of medical devices is a complex and political issue in Benin. The desire of politicians to have equipment sent to their constituency regions is part of a political strategy to gain further electoral support. Furthermore, the preferential distribution of equipment to their region holds potential financial advantages, as they are able to offer more medical examinations or analyses at lower prices to patients, which are not often prescribed for the types of healthcare facilities in their region. For example, color ultrasonic and electrophoresis equipment were found in both rural and urban district health centers (CSC) when zone or departmental hospitals (CHD) would be lacking. Due to its benefits for the reputation of the health facility, hospital directors, managers and users of equipment often fail to report distribution problems, which hinders service delivery in many health facilities.

We found that the degree of power possessed by key actors relative to solving HTM problems was inverse to their perception of the severity of problems. The biomedical, clinical and healthcare technology engineers and technicians appear to face the greatest amounts of problems in HTM, however, their insights are not usually taken into account during policy development. Their dissatisfaction with budget allocation conflicts with the interests of other actors with more political influence. In contrast, the policy-makers, planners and administrators at the MoH have a position of great power relative to solving the problems in HTM, but perceive those problems much less severely. The observation that most interviewees highlighted problems outside their own professional remit supports the argument that the issue is a multilevel system problem. As a consequence, it is pertinent in policy development to identify, at each level, the relevant key actors who are important as enablers towards more effective and efficient HTM processes ie, those actors that recognize the faults of the system and envision an improved system in the future. The alignment of perspectives regarding problems and solutions in HTM would enable a major leap in organizing better healthcare support at the technical level.^40,41^

Differences in the perceptions of problems are also due to conflicts of interest among the workers of the DIEM, which is mainly staffed by construction and civil engineers and has historically been overseen by a civil engineer. Construction and civil engineers have thus been more involved in the planning and financial resource allocation for healthcare facilities than biomedical engineers, meaning the majority of construction and equipment decisions have been under their control. During budget allocation meetings, in the absence of scientific information related to the costs of construction and equipment, an arbitrary financial budget was decided. 25% of the total construction and equipment budget was allocated to equipment procurement, management and maintenance and 75% was allocated for construction works. This sets a perverse incentive for construction and civil engineers to seek the purchase of more costly equipment; benefitting due to the higher costs they are able to charge for the equipment’s placement. The local and foreign suppliers were the second-highest ranked actors, with regards to their ability to solve problems in HTM. Their lobbying and financial power has granted them great influence on policy-makers, however, their financial interests often do not align with the conditions to bring about effective and efficient management of healthcare technologies.

From the findings of the site visits, the prioritized problems in HTM were: “maintenance and repair,” “distribution,” “installation and commissioning,” “use” and “training and personnel skill development.” Many maintenance problems were characterized by the low operational performance of equipment ie, their low rate of availability and ability to function. This could be explained by the perception held by policy-makers that maintenance and repair activities are not a significant problem in Benin. Similar findings have been in reported in Kenya, whereby a decreasing recognition for maintenance and repair capacities aligned with decreasing hospital size.^38^ The lack of maintenance expertise contributes to the high burden of obsolete equipment in healthcare facility wards. In the absence of a technical diagnosis, it is impossible to plan for obsolete equipment, or know if obsolete equipment could be restored to function.

It can be concluded that HTM remains a persistent problem in Benin’s public health system. Many surveys conducted in sub-Saharan African, and other low-income countries – such as Costa Rica, Ghana and Yemen,^15^ Cyprus,^14^ Kenya and South Africa,^38^ Zambia, Botswana and Namibia,^16^ Sri Lanka,^42^ and 144 other countries^9^ – have explored similar issues in HTM, yet have revealed different findings. All these studies have described the symptoms of poor medical equipment performance and ineffective HTM but did not assess the perceptions of key actors with regards to the problems and their power positions relative to solving them.

Previous research has investigated the high acquisition costs of equipment that many actors cited as a problem in HTM.^43,44^ A quantitative analysis investigated the effects of 2 procurement codes that sought to decrease the costs of healthcare technologies, yet this showed that their impact was limited and potentially worsened the situation.^44^ A lack capacity to monitor, or government insight into, supplier prices could have contributed to this.^43^ Additionally, prices may have been increasing due to development in the field of medical devices, or that weaknesses in the government’s public procurement system made it susceptible to corruption. To delineate the 2, public and private sector acquisition price were compared for the same devices and data were controlled for seasonal fluctuations in healthcare technology prices.^44^ This showed that the public sector paid significantly more for healthcare technologies than their private counterparts, and thus increasing prices due to development in the field could not cause this. Equally, procurement prices were greater than could be accounted for by seasonal fluctuations which suggest that suppliers were inflating their margins during public procurement open tenders.

## Limitations and Strengths of the Study

Although separate use of questionnaires and interviews revealed some of the same HTM problems identified by actors, it did not produce consensus on the priority of these problems. For example, the “high acquisition costs and origin of equipment” was ranked as the most pertinent problem during interviews whereas it was ranked fourth in the questionnaires. Additionally, low priority was given to the “high proportion of use of hazardous and unsafe equipment” by both questionnaire respondents and interviewees, although this was a significant sub-topic highlighted during site visits.

The site visits, although providing clear insights into practical HTM problems that healthcare facilities face (especially maintenance related issues), could not shed light on issues such as budgeting, financing, policy, strategic management, planning and procurement, as these are managed by the MoH.

## Further Research

The low priority given to the use of unsafe equipment could be due to the fact that, till now, no performance checks are conducted after the repair of equipment to confirm whether this action has been successful and if the equipment is free of risk, or not. Therefore, further research that investigates the safety of equipment should be conducted to highlight this component of HTM. Additionally, reviewing which types of equipment were out of operation in central and regional healthcare facilities would indicate the state of the situation more accurately. The nonperformance of less common and expensive equipment, such as CT scanners, would yield different considerations for planners and clinicians as opposed to an impairment in one ultrasound machine of many. The main question that arises as a result of this study, is how can changes be made to improve Benin’s HTM system so that STEEEP criteria can be fulfilled? Further exploration into the root causes of problems in HTM will help solutions to be devised that could be incorporated into a HTM policy.

The previous research, cited above, as well as the results and analysis of this study have both been used to inform the content of a policy developed for HTM, validated in April 2016.^45^ Additionally, the DIEM has integrated the policy’s budgeted action plan into their national 5-year financial forecast. The policy has sought to address the problems in HTM reported in this paper, whilst also incorporating the perceptions of a wider diversity of stakeholders during its development. The improvement of Benin’s HTM system is achievable if the key actors in HTM understand the need to align their perspectives, and thus participative and interactive sessions have been used during policy development in an attempt to reconcile the differences in motivations between actors across levels of the HTM system. This helped, in one example, with policy-makers who had previously overlooked the importance of maintenance capacities in healthcare facilities. When contrasted against the perception of international organization representatives and financial donors, however, for whom it had always been a priority, it served to move the issue further up the policy agenda.

These sessions included focus group discussions, interviews and expert meetings, which also serve to make stakeholders in HTM aware of the advantages of a more efficient HTM system and build support for the policy’s aims with their inclusion.

## Conclusion

HTM in Benin’s public health sector faces many problems, which is typified by the unavailability, limited availability or poor state of equipment in many healthcare facilities. The way these problems could be addressed was linked to the priority assigned by key actors in HTM to those problems. Perceptions of the severity of problems by the different actors were inversely related to the political or administrative power they possessed relative to solving the problem. Previous HTM policies have not incorporated the views of low-level actors, even though they face the greatest amount of problems concerning HTM. The lack of experiential and technical knowledge in decision-making and policy development processes could underpin many of the continuing problems in Benin’s HTM system. Before solutions can be devised to these problems in HTM, it is necessary to investigate their root causes, and which problems are most amenable to policy development.

## Acknowledgements

This study was financed by the Netherlands Organization for International Cooperation in Higher Education (NUFFIC), The Hague, The Netherlands, grant numbers NFP-PHD.07/218 and CF3914/2007. Granted to P. Thierry Houngbo for PhD research.

## Ethical issues

The research presented in this article did not require formal approval from a (medical) ethical committee to be conducted. However, during qualitative data collection procedures, ethical considerations were addressed in the following ways: participants were briefed with information about the study; conduct of interviews and their audio recording was based on informed consent; participants could terminate interviews or questionnaires without providing justification, and; unique identification numbers were assigned to responses to protect confidentiality. During all data collection procedures, access to research materials was limited to members of the research team and hard-copy research materials were stored in locked cabinets with restricted access.

## Competing interests

Authors declare that they have no competing interests.

## Authors’ contributions

PTH collected the data under the supervision of DM and LD. PTH, MZ, TDCB, DM, and JB analyzed the data. PTH, MZ, HC, DM, and JB drafted the manuscript which was subsequently checked by all other authors.

## Authors’ affiliations

^1^Ministry of Health, Cotonou, Republic of Benin. ^2^Athena Institute, Vrije Universiteit, Amsterdam, The Netherlands. ^3^Polytechnic School, University of Abomey-Calavi, Cotonou, Republic of Benin.

## Key messages

**Implications for policy makers**

- The actors most frequently rated, during interviews and questionnaires, to possess the political or administrative power necessary to solving problems in healthcare technology management (HTM) in Benin, such as policy-makers and healthcare technology suppliers, were also the least likely to have severe perceptions of those problems. Policy development can be enriched by incorporating the perceptions of a wider diversity of actors, as the technical information they possess, relative to their professional remit, allows them to more easily conceive solutions to problems in the system.
- Problems occurring within an actor’s professional remit were less likely to be identified as such during interviews and questionnaires. Therefore, the chances of improving the HTM system in Benin will be enhanced when differences in perspective across levels of the HTM can be reconciled. It is pertinent for policy-makers to identify, at each level, those actors who can act as enablers towards an improved system ie, actors who recognize the faults of the system and envision a more effective and efficient system in the future.
- The professional demographic of the policy community appears to have influenced which problems in HTM have been historically prioritized in policy content. In Benin, greater representation of civil engineers in the Ministry of Health (MoH) has given them control over a larger proportion of budgets and more influence on health facility planning, as compared to equipment technicians and engineers. It is recommended to develop policy in an evidence-based fashion, ie, using knowledge produced by a diverse group of equally-represented professions and the best empirical information available at the time, as this will help to diminish perceptions that are motivated by financial or professional self-interest.

**Implications for public**

Healthcare technologies offer many benefits to patients, being the most abundant and widely used medical products in practice; however their management has challenges, particularly for sub-Saharan African countries, where many technologies are imported and financial resources are more constrained. In Benin, previous healthcare technology management (HTM) policies have failed to make technologies convert to better quality of care for the population. This research used the perceptions of many stakeholders across the HTM system in Benin to identify its problems and actors with the greatest ability to solve these problems. This may help the public so that problems experienced in healthcare facilities are more commonly identified by future policies, and improved by using the relevant technical knowledge of the right stakeholder to solve the problem. Policies developed with a greater diversity of stakeholders participating may improve accountability in government, and ensure attitudes motivated by self-interest are not able to distort policy agendas.
